# T-Cell Prolymphocytic Leukemia: An Experience from a Tertiary Cancer Centre in South India

**Published:** 2018-04-01

**Authors:** Suresh Babu MC, Abhishek Anand, Kuntegowdanahalli C. Lakshmaiah, Govind Babu K, Dasappa Lokanatha, Linu Abraham Jacob, DS Madhumathi, Kadabur N Lokesh, AH Rudresha, LK Rajeev, Rajesh Patidar

**Affiliations:** Department of Medical Oncology, Kidwai Memorial Institute of Oncology, Bangalore, India

**Keywords:** T-cells, Prolymphocytic leukemia, Alemtuzumab

## Abstract

**Background: **T-cell prolymphocytic leukemia (T-PLL) is a rare lymphoid malignancy with dismal prognosis. Most patients have increased lymphocyte count (>1,00,000/dL) and widespread disease at presentation. Despite high response rate seen with alemtuzumab, the disease relapse is inevitable.

**Materials and** **Methods** ***: ***This was a retrospective observational study done at a tertiary cancer center in South India. All patients diagnosed with T-PLL from August 2010 to July 2015 were studied for the clinical characteristics, pathological findings and treatment outcomes.

**Results** ***: ***Seven patients were diagnosed as T-PLL over a period of 5 years. The median age at diagnosis was 51 years. In the present series, 6 patients (86%) had splenomegaly and 3 had hepatomegaly (43%). Generalized lymphadenopathy was seen in 4 (57%) patients at presentation. Skin lesions were seen in 5 (71%) patients, whereas pleural effusion was seen in only one patient (14%). All had elevated total leukocyte count, with more than 1, 00,000/dL in 4 patients. The median survival was 5 months with different chemotherapy (CT) regimens (5 patients treated with CT and 2 received best supportive care).

**Conclusion:** T-PLL is a rare disease with no definite treatment guidelines. At present, the best outcomes are achieved if treatment with alemtuzumab is followed by stem cell transplant, but the disease invariably relapses. Countries where affordability remains a big challenge, the best approach needs to be defined beyond the monoclonal antibodies and transplant.

## Introduction

 T-cell prolymphocytic leukemia (T-PLL) is a rare lymphoid malignancy which presents at an advanced age and carries a dismal prognosis. The disease is characterized by the proliferation of small- to medium-sized prolymphocytes arising from mature post-thymic T cell ^1^^,^^2^. The disease entity was first described in 1973^1^ and was named variably as diffuse small cleaved cell leukemia, small lymphocytic lymphoma (Working Formulation^3^) and chronic lymphocytic leukemia–T cell type (Kiel classification)^4^. T-PLL was recognized as a distinct entity in 1994 and was clearly separated from other large granular lymphocytic disorders by REAL classification ^5^. Skin infiltration and serous effusions are the common presenting features of T-PLL^2^^,^^6^. Most patients have increased lymphocyte count (>1,00,000/dL) and widespread disease at the time of diagnosis^7^. Compared to B cell prolymphocytic leukemia, anemia and thrombocytopenia are less commonly seen^6^. The prognosis of T-PLL still remains poor with short survival and no curative therapy. The best response has been seen with anti-CD 52 monoclonal antibody alemtuzumab. Despite high response rate, the disease relapse is inevitable^8^^,^^9^. Herein, we studied the clinicopathological profile and treatment outcome in 7 patients with T-PLL treated at a tertiary cancer center in South India.

## MATERIALS AND METHODS

 This was a retrospective observational study done at a tertiary cancer center in South India. The medical records of all patients diagnosed with T-PLL from August 2010 to July 2015 were reviewed to study the clinicopathological profile and treatment outcome. Patient’s clinical characteristics, pathological findings and treatment outcomes were analyzed. The diagnosis of T-PLL was based on the peripheral blood and bone marrow specimen, including immunophenotyping study. 

## Results

**Patients' characteristics**

We found 7 patients of T-PLL over a period of 5 years. The median age at diagnosis of T-PLL was 51 years. Out of 7 patients, 4 were male and 3 female. Two male patients were smoker. Two patients with hypertension were on regular medication. None of the patients gave a history of prior radiation, exposure to chemicals or other malignancies. The history of familial or genetic disorders was negative in all patients. 

**Clinical presentation**

All patients presented with the complaint of easy fatigability with a median duration of 1 month before consultation. The median ECOG performance status was 2. Five out of 7 patients also presented with skin lesions which were in the form of maculopapular rash in 3 and erythematous nodule in 2 patients. Six out of 7 patients had splenomegaly, whereas 3 patients had hepatomegaly. Lymphadenopathy was present in 4 patients at the time of diagnosis. One patient had pleural effusion which was diagnosed during the routine workup. 

**Pathological findings**

Elevated total leukocyte count (> 1,00,000/dL) was seen in 4 patients. Anemia and thrombocytopenia were reported in 2 and 3 patients, respectively. Lactate dehydrogenase (LDH) level was raised in all patients. The mean LDH level was 545. Hyperuricemia was seen in 3 patients at the time of initial evaluation although frank tumor lysis syndrome was not seen in any of them.

Peripheral blood smear showed the presence of atypical lymphoid cells in all patients. Bone marrow aspirate examination was done in all patients. The pathological findings were suggestive of lymphoproliferative disorder with lymphocyte ranging from 80 to 95%. Immunophenotyping (IPT) study confirmed the diagnosis of T-PLL with positive CD5, CD2, CD7 and negative TdT, CD1a. Cytogenetic studies failed to yield metaphases in 4 patients, while complex karyotype was seen in the 3 remaining patients (>3 cytogenetic abnormalities).

**Treatment**

Out of 7 patients, 2 did not opt for any treatment and received best supportive care. One patient received FCM- (fludarabine+cyclophosphamide+mitoxantrone) based treatment, 2 patients were treated with CHOP (cyclophosphamide+doxorubicin+vincristine+prednisolone), and another two were given chlorambucil plus prednisolone (Table. 1). Due to financial constraints, none of our patients received alemtuzumab. Complete response was seen in the patient who received FCM and transient partial response was seen in one patient treated with CHOP. The median survival was 5 months. The patient receiving FCM- based regimen is still alive and is under regular follow-up. Treatment outcomes are shown in Table 1. 

**Table 1:** Treatment outcome

**S.No**	**Age**	**Treatment ** **received**	**PR**	**CR**	**Complication ** **of treatment**	**Survival ** **(months)**
1	45	FMC	-	+		12(alive)
2	51	NA	-	-		2
3	42	Chl+P	-	-		8
4	59	CHOP	+	-		7
5	55	NA	-	-		5
6	47	CHOP	-	-	FN	3
7	61	Chl+P	-	-		4

Chl+P – Chlorambucil + Prednisolone, CHOP – Cyclophosphamide +Doxorubicin + Vincristine + Prednisolone, CR – Complete Response, FCM – Fludarabine+Mitoxantrone + Cyclophosphamide, FN – Febrile Neutropenia, PR – Partial Response

## Discussion

 T-PLL is a rare hematological malignancy and represents less than 2% of mature lymphocytic leukemias ^1^^,^^7^^,^^10^. It has been considered a disease of elderly men with an average age of 65 years ^11^. In our series, the median age at presentation was 51 years, which is comparatively less than other published studies. The male to female ratio was 1.3:1 in our study. Most of the patients presented with splenomegaly (82-92%), hepatomegaly and generalized lymphadenopathy ^10^. Skin lesions were present in 27%, whereas pleural effusion was seen in 14% of patients at presentation ^7^. In the present series, 6 patients (86%) had splenomegaly, 3 had hepatomegaly (43%) and generalized lymphadenopathy was seen in 4 (57%) patients. Skin lesions were seen in 5 (71%) patients, whereas pleural effusion was found in only one patient (14%). The incidence of the skin lesion in our series was much higher than previously described in different studies.

Anemia and thrombocytopenia are seen in half of the patients with T-PLL. In the present study, anemia and thrombocytopenia were present in 2 (28.6%) and 3(43%) patients, respectively. Peripheral blood examination usually shows marked lymphocytosis with lymphocyte counts frequently more than 1 lakh ^1^. In our study, total leukocyte count of more than one lakh was seen in 4 (57%) patients. More than 90% of the circulating cells are prolymphocytes. The prolymphocytes are typically medium sized with condensed chromatin, single prominent nucleolus and intensely basophilic nongranular cytoplasm with cytoplasmic protrusions or blebs. The nuclei may be round, oval or irregular in shape ^12^^,^^13^.

There is diffuse infiltration of bone marrow by prolymphocytes. In our series, examination of bone marrow revealed the presence of lymphoproliferative disease with more than 80% lymphocytes in all patients. Diagnosis of T-PLL is made by flow cytometry which can be done either on peripheral blood or bone marrow sample. The monoclonal lymphocyte population is positive for T- cell markers, including CD2, CD3, CD5 and a strong CD7. There is a variable expression of CD4 and CD8. T-cell prolymphocytes are mature post-thymic peripheral T- cell and do not express TdT and cortical thymic marker CD1a. CD52 is expressed at high density, which can be used for targeted therapy with alemtuzumab ^11^^,^^14^. CD26 and TCL-1 protein expression are the most specific markers for T-PLL and the overexpression of TCL-1 oncogene can be useful in detecting residual T-PLL after therapy^15^. In our series, all patients were positive for CD2, CD5, CD7 and negative for TdT and CD1a. CD4+CD8- was seen in 4 (57%); CD4+CD8+ in 2(29%) and CD4-CD+ in 1(14%) patient. 

T-PLL are often resistant to therapy and have an aggressive course. With conventional regimens, the response rate is around 30% and the overall survival remains poor with a median survival of 7 months ^7^. Alemtuzumab is now the first-line treatment of T-PLL which is effective and well tolerated. The overall response rate ranges from 51-95% with a median survival of 15 – 19 months in those who achieve complete response^16^^-^^18^. The use of consolidation treatment after stem cell transplantation increases long-term relapse-free survival ^19^^,^^20^. The purine- analog fludarabine has been tried in combination with cyclophosphamide and mitoxantrone (FMC) with an ORR rate of 68% ^21^.

In the study done by Farhad Ravandi et al. ^22^, none of the 8 patients showed CR with fludarabine-based treatment. In contrast, the present study showed that only one patient treated with FMC achieved CR. This may suggest a role of fludarabine-based chemotherapy in patients with T-PLL. It might achieve a better outcome if many patients could afford the standard therapy including alemtuzumab and consolidation with stem cell transplantation.

## CONCLUSION

 T-PLL is a rare disease with no definite treatment guidelines. Although alemtuzumab and stem cell transplantation have been the standard treatment at present, cost remains a major challenge. Moreover, purine analogs like fludarabine-based regimens may show a good promise in the management of T-PLL. Larger studies are needed for optimizing the treatment protocols for this rare malignancy.
